# Editorial to “Atropine sulfate may be effective to recover the unstable hemodynamics in coronary artery spasms related to atrial fibrillation ablation procedures”

**DOI:** 10.1002/joa3.13102

**Published:** 2024-06-25

**Authors:** Yuichi Hori, Hideyuki Aoki, Shiro Nakahara

**Affiliations:** ^1^ Department of Cardiology Dokkyo Medical University Saitama Medical Center Koshigaya Japan

**Keywords:** atrial fibrillation, atropine sulfate, autonomic nervous system, catheter ablation, coronary artery spasm

Editorial comment on “Atropine sulfate may be effective to recover the unstable hemodynamics in coronary artery spasms related to atrial fibrillation ablation procedures.”^1^

The utility of pulmonary vein isolation (PVI) as an initial therapy for patients with atrial fibrillation (AF) has been established. The elimination of PV electrical firing was initially targeted to prevent AF occurrence, however, the achievement of high therapeutic outcomes led to focusing on the secondary effects of the PVI such as modification of imbalances of the autonomic nervous system. Simultaneously, the need to control an overreaction of the autonomic nervous reflex during the PVI has been reported. In particular, excessive activity of the parasympathetic nervous system is considered one of the pathological causes of coronary artery spasms (CASs) during the PVI, which may also cause unstable hemodynamics. In this issue, Kawai et al. report interesting cases of the use of atropine sulfate in CAS patients with hemodynamic instability during AF ablation.^1^

The autonomic nervous system is maintained by the balance of the sympathetic and parasympathetic nervous systems and is controlled by both nervous systems activating together in an optimal tone. Therefore, a precise interpretation of the autonomic nervous tone is complex and is difficult to control just by the hemodynamic information. Regarding the case of CASs with unstable hemodynamics, an excessive tone of the parasympathetic response is expected, however, how the sympathetic nervous system is reacting is obscure. As suggested by Kawai et al., the use of atropine sulfate would simply block the excessive parasympathetic activation and lead to a remarkable recovery of the hemodynamics. Although the direct effect of atropine sulfate in relieving CASs is not proven and requires further study, its usefulness in maintaining the patient's condition is noteworthy.

The occurrence of CASs during the PVI has been reported by Nakamura et al. and was 0.19% among 22,232 patients.^2^ Fifty percent of the CASs were observed during the PV ablation, and the left superior pulmonary vein (LSPV) was the most frequent site. Those results were considered to be the effects of the epicardial ganglion plexus (GP) located at the periphery of the PVs, which is strongly innervated by the parasympathetic nervous system.^3^ In addition, they highlighted that 17% (7/42) of CASs result in a serious condition, such as ventricular fibrillation or cardiopulmonary arrest, requiring cardiopulmonary resuscitation. In those cases, the onset of an uncontrollable autonomic nervous condition is expected to contribute to the failure of a spontaneous recovery and to progress to an unstable hemodynamic state. As mentioned previously, the autonomic nervous system is controlled continuously by having the sympathetic and parasympathetic nervous systems compensate. Therefore, to interpret these CASs and various conditions, the factor that causes the imbalance of the autonomic nervous system must be considered.

The two cases of CASs that Kawai et al. reported occurred when the sheath passed through the septum, and when pulled out from the atrial septum.^1^ Hachisuka et al. reported a perioperative CAS in AF ablation, and consistent with the report of Nakamura et al., the time when some mechanical stress was applied to the septum was one of the common situations.^4^ Both sympathetic and parasympathetic postganglionic nerve fibers innervate this aspect of the atrial septum, including the AV node. Mechanical stress to this area may have provoked a strong parasympathetic reflex, while how the sympathetic nervous system responded was difficult to determine. However, since spontaneous recovery was not expected in two cases, it is likely that the attenuation of a sympathetic response or an extremely strong parasympathetic response that could not be balanced may have occurred. The use of thiopental and persistent AF is reported as a factor of a severe condition during CASs.^2^ Thiopental directly activates the parasympathetic system and attenuates the sympathetic nervous system, and persistent AF patients have been reported to have a stronger vagal response to GP stimulation than paroxysmal AF.^5^ The cases reported by Kawaii et al. were both symptomatic paroxysmal AF patients, and the use of thiopental was not mentioned. Therefore, the pathological condition of CASs during AF ablation may be much more complicated and need further investigation.

The occurrence of CASs during AF ablation is rare, however, both Nakamura et al. and Hachisuka et al. concluded in their reports that you must always be aware of the potential for CASs throughout the procedure.^2^, ^4^ The infusion of nitroglycerin would be the first choice for relieving the CASs, while as Kawai et al. demonstrated, a combined use of atropine sulfate, which simply blocks the excessive parasympathetic activation, may prevent a fatal situation in some cases. As with conventional thermal ablation devices, there are increasing reports on the generation of CASs and the neural effects of pulsed‐field ablation. We should always be aware of this complication during AF ablation and also be prepared for fatal situations.

## CONFLICT OF INTEREST STATEMENT

Authors declare no conflict of interests for this article.

## References

[1] Kawai S , Okahara A , Tokutome M , Matsuura H , Mukai Y . Atropine sulfate may be effective to recover the unstable hemodynamics in coronary artery spasms related to atrial fibrillation ablation procedures. J Arrhythmia. 2024. 10.1002/joa3.13090 PMC11317650 39139870

[2] Nakamura T , Takami M , Fukuzawa K , Kiuchi K , Kono H , Kobori A , et al. Incidence and characteristics of coronary artery spasms related to atrial fibrillation ablation procedures—large‐scale multicenter analysis. Circ J. 2021;85(3):264–271. 33431721 10.1253/circj.CJ-20-1096

[3] Tan AY , Li H , Wachsmann‐Hogiu S , Chen LS , Chen PS , Fishbein MC . Autonomic innervation and segmental muscular disconnections at the human pulmonary vein‐atrial junction: implications for catheter ablation of atrial‐pulmonary vein junction. J Am Coll Cardiol. 2006;48(1):132–143. 16814659 10.1016/j.jacc.2006.02.054

[4] Hachisuka M , Fujimoto Y , Oka E , Hayashi H , Yamamoto T , Murata H , et al. Perioperative coronary artery spasms in patients undergoing catheter ablation of atrial fibrillation. J Interv Card Electrophysiol. 2022;64(1):77–83. 34773218 10.1007/s10840-021-01089-6 PMC9236998

[5] Iso K , Okumura Y , Watanabe I , Nagashima K , Takahashi K , Arai M , et al. Is vagal response during left atrial ganglionated Plexi stimulation a Normal phenomenon?: comparison between patients with and without atrial fibrillation. Circ Arrhythm Electrophysiol. 2019;12(10):e007281. 31610720 10.1161/CIRCEP.118.007281

